# SARS-CoV-2 infection in 3,241 School working staffs: Impact of SARS CoV-2 variants of concern [Wild, B.1.1.7 and Omicron]

**DOI:** 10.1371/journal.pone.0291989

**Published:** 2023-10-04

**Authors:** Moza Alishaq, Jameela Ali Al Ajmi, Mohammed Shaheen, Mohamed Elgendy, Suni Vinoy, Anil George Thomas, Sam Joseph, Tintu Elizabeth Mathew, Renjith Joseph, Christymol Thomas, Anju K. Alex, Bincy Thomas, Asmaa Nafady, Hamed Elgendy, Hanaa Nafady-Hego

**Affiliations:** 1 Corporate Quality Department, Hamad Medical Corporation, Doha, Qatar; 2 Faculty of Medicine, Universiti Sains of Malaysia, Kelantan, Malaysia; 3 Faculty of Medicine, Clinical and Chemical Pathology Department, South Valley University, Qena, Egypt; 4 Anesthesia Department, Hamad Medical Corporation, Doha, Qatar; 5 Anesthesia Department, Weill Cornell Medical College, Doha, Qatar; 6 Faculty of Medicine, Anesthesia Department, Qatar University, Doha, Qatar; 7 Faculty of Medicine, Microbiology and Immunology Department, Assiut University, Assiut, Egypt; 8 Al Tahrir Medical Center, Doha, Qatar; Zagazig University Faculty of Human Medicine, EGYPT

## Abstract

**Background:**

There is debate over whether physical attendance at school affects the spread of the SARS-CoV-2 pandemic.

**Methods:**

A cohort of personnel from several schools in Qatar provided nasopharyngeal swabs (NPS) for SARS-CoV-2 RT-PCR and rapid antigen testing. Each of them was monitored for infection until February 2022.

**Results:**

In total, 3,241 employees gave samples for analysis. Prior to the start of the 2020–2021 academic year (Group I), 3.49% of samples tested positive for SARS-CoV-2. Most of the positive PCR results were from male, senior, non-teaching staff members. Only 110 (3.39%) employees who had enrolled in face-to-face instruction before the B.1.1.7 variant’s emergence (Group II), 238 (7.34%) after the B.1.1.7 variant’s emergence (Group III), and 410 (12.65%) after the introduction of the Omicron variant (Group IV) had reported infection by PCR test. Most people who tested positive by PCR after enrolling in school were young, female teachers. In the Cox Proportional-Hazards Model, exposure to a confirmed case, the presence of symptoms in the two weeks prior to exposure in all groups—young age in Groups II and III, male gender in Groups I and IV, shared housing in Group III, and the presence of comorbidities in Groups II and III independently predicted SARS-CoV-2 infection in school staff.

**Conclusion:**

Critical information about the risk of SARS-CoV-2 infection in school workers during the whole pandemic is provided by our study. School operations in Qatar were made safer through initial and ongoing screenings, as well as widespread vaccination of school personnel.

## Introduction

The higher authorities’ attention was drawn to the timing of the reopening of schools with full in-person attendance after the SARS-CoV-2 pandemic first appeared in Qatar on February 29, 2020. Because of the significant physical, psychological, and financial strain on families as well as Qatar’s preference to benefit from human interaction, they permitted opening with full in-person attendance at various phases [1–7]. Although some people thought that delaying in-person attendance indefinitely was necessary due to the ongoing pandemic’s increasing case counts, hospitalizations, and fatality rates among infected persons, as well as the probable increased involvement of children in the pandemic’s spread [8–12]. The state of Qatar implemented a number of measures to reduce the rate of viral transmission in schools when it opened. All school staff members underwent a systematic testing and screening method at the start of the academic year, followed by ongoing testing that successfully identified most probable illness transmitters. Identification and isolation of such individuals, widespread immunization of all schoolwork personnel, and a weekly request for students to submit a negative result to a rapid antigen test were all implemented. In addition to tight mask use and physical distancing restrictions for all staff, students, and attendants dropping off or picking up students, hybrid learning for students with a limit of 30–50% attendance on any given day on a rotational basis was also implemented. There is proof that transitioning from in-person to online classrooms increases dropout rates among students, which has an impact on their development and performance [7]. Additionally, I believe that the overwhelming body of information demonstrating that schools did not contribute to the spread of COVID-19, at least among students [6,13]. According to reports from the authorities, three significant variations of concern afflicted Qatar: the wild variant [28-02-2020 to 24-12-2020]; the B.1.1.7 variant [25-12-2020 to 16-12-2021]; and the Omicron variant [on 17-12-2021].

Our study’s goal is to characterize the baseline prevalence of SARS-CoV-2 infection and incidence of infection after school reopened, as well as during the 3 varieties of concern among the schools’ workers, by describing a portion of the Qatari experience.

## Methods

### Study participants and populations

A retrospective observational cohort study that was conducted in the period from February 28, 2020, till February 20, 2022 in Qatar. The first academic year in Qatar following the onset of the SARS-CoV-2 pandemic began on August 23, 2020, for the 2020–2021 academic year. All school staff in Qatar participated in a nationwide testing and screening campaign that included providing a nasopharyngeal swab [NPS] for RT-PCR testing for the SARS-CoV-2 virus. Then, throughout the pandemic, several interventions were put in place in all schools to lessen the risk of infection transmission. They included strict mask use and physical distancing policies for all staff, students, and attendants dropping off or picking up students, requiring all staff to have a negative NPS for SARS-CoV-2 by PCR at the beginning of the school year, allowing hybrid learning for students with a maximum of 30–50% attendance on any given day, and requiring hybrid learning for students with no more than 80% attendance. All school workers are required to have full vaccination or present a negative RT-PCR result on a weekly basis, school students should present a negative rapid antigen test on a weekly basis or be vaccinated [14]. To understand the dynamics of infection among school employees and the effects of variants of concern on that population, 3,241 school staff members were monitored by SARS-CoV-2 for two academic years using RT-PCR or rapid antigen. Testing was performed for contact tracing, symptoms, and non-vaccinated staff. As detailed in our earlier investigations, all testing was carried out at a single national reference laboratory using established commercial assays [14–17]. The study was carried out in accordance with the most recent Helsinki update, and Hamad Medical Corporation’s Institutional Review Board gave its approval. [MRC- 01-20-982]. Since testing was conducted as part of a nationwide testing effort in response to a national health emergency, informed consent was waived.

### Statistical analysis

The study groups were compared using the Mann-Whitney U test, and quantitative data was presented as median and IQR [interquartile range]. The chi-square test was used to compare qualitative data that was presented as frequency and percentage with categorical data. Cox- Hazard regression analysis was used to identify the risk of infection. The variables with a p-value less 0.1 were introduced in the multivariable model. P<0.005 served as the threshold for statistical significance. The data were examined using the Statistical Package for Social Sciences [IBM-SPSS 21]. [SPSS: An IBM Company, version 21.0, IBM Corporation, Armonk, NY, USA].

## Results

From the beginning of the pandemic on February 29, 2020, to February 20, 2022, 3,241 school employees were tested using the SARS-Cov-2 RT-PCR to determine the dynamics of the SARS-Cov-2 infection. Our research period lasted for about two school years. The majority of the study’s participants were middle-aged, with an average age in their mid-30s. More than half of the participants were women. In total, 1,985 women participated in the survey, of whom 1,438 worked as teachers. The study was conducted irrespective of the nationalities, and they were arranged according to the larger number into India, the Philippines, Nepal, Sri Lanka, Egypt, Kenya, Jordan, Bangladesh, and others. Indians made up 42.4% of the participants, while people from the Philippines made up 12%. More than 50% of participants were teachers. Of the 3,241 subjects, 846 cases became positive over time, of which 497 were teachers and 117 had a history of contact with confirmed cases. 79.9% of the participants in the research had no comorbid conditions (Table 1).

**Table 1:** Baseline characteristics of the teaching and non-teaching school staff.

	Total N = 3241	Teaching staff N = 1878	Non-teaching staff N = 1363	p-value
Median age (IQR), years	37 (32,44)	38 (33, 44)	37 (31,44)	0.09
Female sex, N (%)	1985 (61.2)	1438 (76.6)	547 (40.1)	<0.001
Nationality, N (%)				<0.001
Indian	1374 (42.4)	994 (52.9)	380 (27.9)	
Filipino	389 (12.0)	230 (12.2)	159 (11.7)	
Nepalese	365 (11.3)	4 (0.2)	361 (26.5)	
Sri Lankan	255 (11.3)	128 (6.8)	127 (9.3)	
Egyptian	160 (4.9)	119 (6.3)	41 (3.0)	
Kenyan	81 (2.5)	21 (1.1)	60 (4.4)	
Jordanian	63 (1.9)	51 (2.7)	12 (0.9)	
Bangladeshi	54 (1.7)	3 (0.2)	51 (3.7)	
Others	500 (15.4)	328 (17.5)	172 (12.6)	
Type of accommodation, N (%)				<0.001
Single	393 (12.1)	335 (17.8)	58 (4.3)	
Shared	1249 (38.5)	277 (14.7)	972 (71.3)	
Family	1599 (49.3)	1266 (67.4)	333 (24.4)	
Job category, N (%)				<0.001
Teachers	1878 (57.9)	1878 (100)	N/A	
Administrative and support staff	316 (9.8)	N/A	316 (23.2)	
Transportation and security staff	295 (9.1)	N/A	295 (21.6)	
Technician/conductor/laboratory workers	294 (9.1)	N/A	294 (21.6)	
Service staff	458 (14.1)	N/A	458 (33.6)	
Education years, N (%)				<0.001
>16	2186 (67.4)	1847 (89.3)	339 (24.9)	
12–15	294 (9.1)	31 (1.7)	263 (19.3)	
8–11	576 (17.8)	NA	576 (42.3)	
0–7	185 (5.7)	NA	185 (13.6)	
PCR positive, N (%)	846 (26.1)	497 (26.5)	349 (25.6)	0.58
Median (IQR) Ct Values	22 (18, 28)	22(18, 27)	23 (18, 29)	
History of contact with confirmed cases, N (%)	117 (3.6)	73 (3.9)	44 (3.2)	0.32
clinical symptoms, N (%)				<0.001
No	2579 (79.6)	1444 (76.9)	1135 (83.3)	
1–2	434 (13.4)	292 (15.5)	142 (10.4)	
≥3	227(7.0)	142 (7.6)	85 (6.2)	
Co morbidities, N (%)				<0.001
No	2588 (79.9)	1453 (77.4)	1135 (83.3)	
1–2	568 (17.5)	372 (19.8)	196 (14.4)	
≥3	84 (2.6)	53 (2.8)	31 (2.3)	
Type of vaccination, N (%)				<0.001
Not vaccinated	224 (6.9)	87 (4.6)	137 (10.1)	
SARS-CoV-2 (Pfizer)	2805 (86.5)	1696 (90.3)	1109 (81.4)	
SARS-CoV-2 (Moderna)	206 (6.4)	93 (5.0)	113 (8.3)	
SARS-CoV-2 (AstraZeneca)	6 (0.2)	2 (0.1)	4 (0.3)	

Teachers and non-teaching employees each had at least one positive PCR result during the study period, and around 80% of the infected patients were discovered accidentally in surveillance programs without any symptoms (Table 1).

Cases were divided into four groups based on the time when they receive positive results in order to study the effects of school opening and the era of time of infection: Group I [get positive before the start of the first academic year, where the wild variant is dominate [28-02-2020 to 22-08-2020]; Group II, where the wild variant is dominate after school opening [23-08-2020 to 24-12-2020]; Group III, where the B.1.1.7, UK variant is dominate [25-12-2020 to 16-12-2021]; and Group IV, where the Omicron is dominate [17-12-2021to 20-2-2022]. The infection changes with time, being lowest in Groups I and II before increasing in Group III and peaking during the Omicron era [Group IV]. 113 [3.49%] and 110 [3.39%] of the employees in Group I and Group II who were monitored during the 28-02-2020 to 24-12-2020 period while the wild version was circulating became positive.

On December 25, 2020, the coronavirus variant B.1.1.7 was discovered for the first time in Qatar. Group III comprised 238 (7.31%) positive participants were identified and one of them was reinfection.

**Fig 1:**
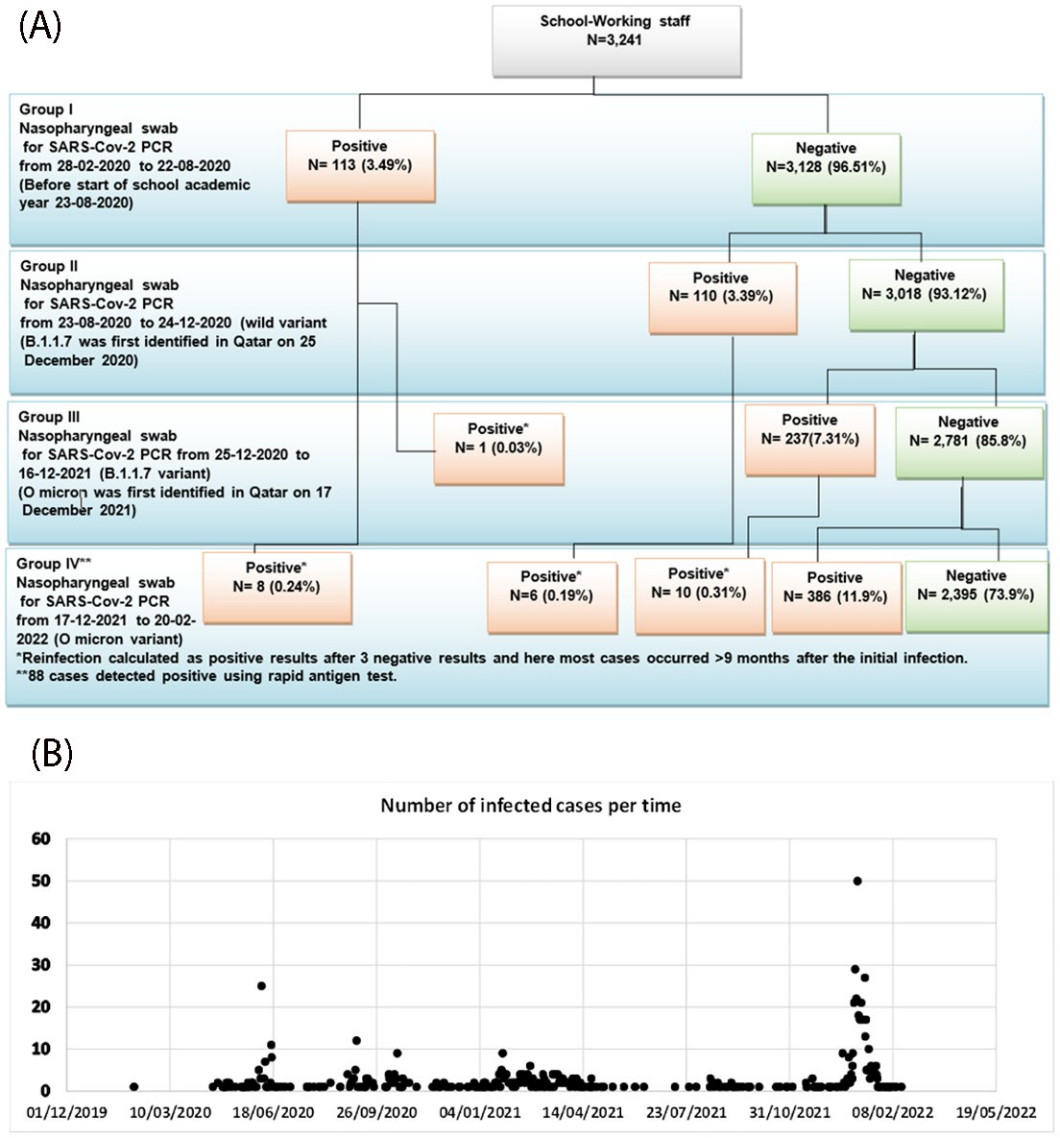
A. Study flowchart. B. Baseline and follow-up PCR testing among all school staff.

Omicron was discovered for the first time in Qatar on December 17th, 2021, and Group IV was investigated from December 17, 2021, to February 20, 2022, the end of the study period, during which time 8 people from Group I, 6 participants from Group II, and 10 participants from Group III experienced re-infection. 386 out of the 2,781 people who tested negative for the Omicron variation turned positive, or 11.9% of the whole population (Fig 1A and 1B).

The infected Group II cases were considerably older. It’s noteworthy to observe that group II, III, and IV infections affect women more frequently than they do men (Table 2).

**Table 2:** Characteristics of study participants identified by PCR-SARS-CoV-2 positivity and or rapid antigen test.

	Total N = 846	Group I N = 113	Group II N = 110	Group III N = 237	Group IV** N = 386	p-value
Median (IQR) Ct Values	22 (18, 28)	23 (18, 29)	22 (18, 29)	22 (18, 29)	22(18, 27)	>0.05
**Median age (IQR), years**	37 (32, 43)	40 (32, 49)	35 (30, 41)	37 (32, 42)	37 (32, 42)	*
**Female sex, N (%)**	510 (60.3)	31 (27.4)	68 (61.8)	171 (72.2)	240 (62.2)	<0.001
**Nationality, N (%)**						<0.001
Indian	333 (39.4)	57 (50.4)	46 (41.8)	114 (48.1)	116 (30.1)	
Filipino	113 (13.4)	6 (5.3)	9 (8.2)	18 (15.9)	80 (20.7)	
Nepalese	87 (10.3)	20 (17.7)	31 (28.2)	19 (8.0)	17 (4.4)	
Sri Lankan	43 (5.1)	7 (6.2)	3 (2.7)	19 (8.0)	14 (3.6)	
Egyptian	68 (8.0)	9 (8.0)	5 (4.5)	21 (8.9)	33 (8.5)	
Kenyan	19 (2.2)	3 (2.7)	1 (0.9)	3 (1.3)	12 (3.1)	
Jordanian	30 (3.5)	1 (0.9)	3 (2.7)	8 (3.4)	18 (4.7)	
Bangladeshi	7 (0.8)	1 (0.9)	2 (1.8)	2 (0.8)	2 (0.5)	
Others	146 (17.3)	9 (8.0)	10 (9.1)	33 (13.9)	94 (24.4)	
**Type of accommodation, N (%)**						<0.001
Single	97 (11.5)	6 (5.3)	10 (9.1)	22 (9.3)	59 (15.3)	
Shared	298 (35.2)	73 (64.6)	63 (57.3)	71 (30.0)	91 (23.6)	
Family	451 (53.3)	34 (30.1)	37 (33.6)	144 (60.8)	236 (61.1)	
**Job category, N (%)**						<0.001
Teachers	497 (58.7)	33 (29.2)	51 (46.4)	158 (66.7)	255 (66.1)	
Administrative and support staff	96 (11.3)	13 (11.5)	24 (10.1)	24 (10.1)	48 (12.5)	
Transportation and security staff	57 (6.7)	34 (30.1)	8 (3.4)	8 (3.4)	6 (1.6)	
Technician/conductor/laboratory workers	66 (7.8)	13 (11.5)	13 (11.8)	18 (7.6)	22 (5.7)	
Service staff	130 (15.4)	20 (17.7)	26 (23.6)	29 (12.2)	55 (14.2)	
**Education years**, N (%)						<0.001
≥ 16	597 (70)	43 (38.1)	63 (57.3)	183 (77.2)	308 (79.8)	
12–15	69 (8.2)	10 (8.8)	10 (9.1)	16 (6.8)	33 (8.5)	
8–11	134 (15.8)	41 (36.3)	29 (26.4)	29 (12.2)	35 (9.1)	
0–7	46 (5.4)	19 (16.8))	44 (3.2)	9 (3.8)	10 (2.6)	
**History of contact with confirmed cases**, N (%)	111 (13.1)	30 (26.5)	21 (19.1)	44 (18.6)	16 (4.1)	<0.001
**clinical symptoms before diagnosis, N (%)**						0.001
1–2	232 (27.4)	41 (36.3)	39 (35.5)	70 (29.5)	82 (21.2)	
≥3	166 (19.6)	29 (25.7)	16 (14.5)	75 (31.6)	46 (11.9)	
**Co morbidities before diagnosis, N (%)**						<0.001
1–2	222 (26.2)	28 (24.8)	29 (26.4)	87 (36.7)	78 (20.2)	
≥3	27 (3.2)	6 (5.3)	8 (3.4)	11 (2.8)		
**Cases with Effective vaccination (≥ 14 days after second dose of vaccination,**	445 (52.6)	NA	NA	66 (27.8)	379 (98.2)	

Group I (get positive before the start of the first academic year; where wild variant is dominate; 23-08-2020 to 24-12-2020); Group II where wild variant is dominate after school opening; Group III where B.1.1.7, UK variant is dominate; and Group IV where Omicron is dominate (17-12-2021-20-2-2022).

* p-value Group I vs. Group II, Group III and Group IV is 0.001, 0.043 and 0.23 respectively; p-value Group II vs. Group III and Group IV is <0.0001 and 0.001 respectively, p-value Group III vs. Group IV is 0.033.

**88 cases in Group IV were detected positive using rapid antigen test.

A total of 289 (35.2%) cases of infection lived in communal quarters and were primarily affected during Groups I and II. Teachers were most likely to become infected, particularly in Groups 3 and 4, hence university graduates made up many infected cases. Only 13.1% of cases had a definite history of interaction with COVID-19 cases that had been confirmed, and most of these contacts occurred during the first term of the school year. In terms of comorbidities, over half of the infected cases had at least one.

Because most participants were immunized at the time, it’s possible that Group IV’s high rate of breakthrough infection was among those who had received vaccinations at the time (Table 2).

In Cox Hazard regression analysis, male gender [HR 3.0, 95% CI (1.9–4.9), p-value <0.001], lower education level [compared to ≥ 16 years of education [8–11 years of education [HR 5.4, 95% CI (1.8–15.8)] and 0–7 years of education [HR 5.5, 95% CI (1.9–16.3)], p-value = 0.002]. The non-teaching profession did not increase the probability of infection, contact with a confirmed case [HR 4.2, 95% CI (2.6–6.9), p-value <0.001], and presence of symptoms in the preceding 2 weeks [1–2 symptoms [HR 6.6, 95% CI (14.1–10.6)], 3 or more symptoms [HR 10.8, 95% CI (6.2–18.9), p-value <0.001]. Independently predicted SARS-CoV-2 infection [PCR or rapid antigen positive] in school staff before school started. In addition, younger age [HR 0.7, 95% CI (0.6–0.9), p-value = 0.01] contact with a confirmed case [HR 3.8, 95% CI (2.2–6.6), p-value <0.001], presence of symptoms in the preceding 2 weeks [1–2 symptoms [HR 3.5, 95%CI 2.2–5.5, p-value <0.001], [3 or more symptoms [[HR 2.2, 95%CI 1.3–4.7, p-value = 0.004]] and presence of comorbidities [1–2 comorbidities [HR 1.8, 95%CI 1.1–3.0, p-value = 0.01] independently predicted SARS-CoV-2 infection [PCR or rapid antigen positive] in school staff after school started and before the emergence of the B.1.1.7, UK variant, in Qatar. Furthermore, younger age [HR 0.8, 95% CI (0.7–0.9), p-value = 0.004], sharing accommodation [shared with non-family member [HR 1.7, 95% CI 1–3.0, p-value = 0.05], [shared with family member [[HR 1.6, 95% (CI 1.0–2.6), p-value = 0.04]], contact with a confirmed case [HR 2.5, 95% CI (1.7–3.5), p-value <0.001], presence of symptoms in the preceding 2 weeks [1–2 symptoms [HR 3.3, 95% CI (2.4–4.6)], [3 or more symptoms [[HR 6.2, 95% CI (4.4–8.9)], p-value <0.001] and presence of comorbidities [1–2 comorbidities [HR 2.3, 95% CI (1.7–3.1), p-value <0.001] independently predicted SARS-CoV-2 infection [PCR or rapid antigen positive] in school staff after the emergence of the B.1.1.7, UK variant, in Qatar. Lastly, male gender [HR 1.3, 95% CI (1.0–1.6), p-value = 0.02], working in the service sector [HR 3.5, 95% CI (1.8–6.9), p-value <0.001], presence of symptoms in the preceding 2 weeks [1–2 symptoms [HR 2.0, 95% CI (1.6–2.6)], [3 or more symptoms [[HR 2.1, 95% CI (1.5–2.8)], p-value <0.001] and presence of comorbidities [1–2 comorbidities [HR 2.3, 95% CI (1.7–3.1), p-value <0.001] independently predicted SARS-CoV-2 infection [PCR or rapid antigen positive] in school staff after the emergence of Omicron variant in Qatar (Table 3).

**Table 3:** Factors associated with SARS-CoV-2 infection in school staff (PCR positive and/or rapid antigen positive).

	Group I N = 113		Group II N = 110		Group III N = 237		Group IV* N = 386	
	HR (95% CI)	p-value	HR (95% CI)	p-value	HR (95% CI)	p-value	HR (95% CI)	p-value
**Age 10-time increase**	1.0 (0.8–1.3)	0.73	0.7 (0.6–0.9)	0.01	0.8 (0.7–0.9)	0.004	1.0 (0.9–1.1)	0.69
**Gender (comparator: female)**	3.0 (1.9–4.9)	<0.001	1 (0.6–1.6)	0.99	0.8 (0.6–1.1)	0.1	1.3 (1.0–1.6)	0.02
**Nationality (comparator: Indian)**								
Filipino	0.8 (0.3–1.9)	0.61	0.7 (0.4–1.5)	0.43	0.5 (0.3–0.9)	0.02	2.2 (1.7–2.9)	<0.001
Nepalese	0.9 (0.5–1.6)	0.66	2.7 (1.3–5.5)	0.01	0.9 (0.5–1.7)	0.80	0.8 (0.5–1.4)	0.49
Sri Lankan	0.4 (0.2–1.0)	0.04	0.4 (0.12–1.3)	0.12	1.1 (0.7–1.9)	0.85	0.7 (0.4–1.3)	0.26
Egyptian	1.9 (0.9–3.9)	0.09	0.9 (0.3–2.3)	0.82	0.6 (0.2–1.7)	0.78	2.3 (1.6–3.3)	<0.001
Kenyan	1.5 (0.4–5.4)	0.56	0.4 (0–2.9)	0.35	0.6 (0.2–1.9)	0.36	1.8 (1.0–3.3)	0.07
Jordanian	0.4 (0.1–3.1)	0.39	1.1 (0.3–3.6)	0.88	0.9 (0.4–1.9)	0.84	3.3 (2.1–5.3)	<0.001
Bangladeshi	0.4 (0–2.7)	0.32	1.4 (0.3–6.7)	0.64	0.9 (0.2–3.9)	0.90	0.7 (0.2–2.1)	0.48
Others	0.6 (0.3–1.2)	0.14	0.6 (0.3–1.2)	0.12	0.7 (0.4–1)	0.04	2.2 (1.6–2.8)	<0.001
**Type of accommodation (comparator: single)**								
Shared	2.0 (0.8–5.0)	0.16	1.9 (0.9–4.1)	0.09	1.7 (1–3.0)	0.05	0.6 (0.4–0.9)	0.2
Family	1.4 (0.6–3.5)	0.44	0.9 (0.5–1.9)	0.82	1.6(1.0–2.6)	0.04	0.9 (0.7–1.2)	0.62
**Job category (comparator: Teachers)**								
Administrative and support staff	1.2 (0.6–2.6)	0.57	1.0 (0.4–2.9)	0.93	0.8 (0.5–1.3)	0.41	1.2 (0.8–2.0)	0.42
Transportation and security staff	1.2 (0.4–3.9)	0.73	1.1 (0.3–3.7)	0.91	0.6 (0.2–1.6)	0.29	0.5 (0.2–1.4)	0.19
Technician/conductor/laboratory workers	0.6 (0.2–1.7)	0.32	1.2 (0.6–2.5)	0.55	0.9 (0.5–1.8)	0.85	1 (0.7–1.4)	1
Service staff	0.7 (0.2–2.3)	0.56	1.3 (0.4–4.2)	0.63	1.2 (0.5–1.3)	0.64	3.5 (1.8–6.9)	<0.001
**Education years (comparator: ≥ 16)**								
12–15	2.1 (0.80–5.8)	0.13	0.84 (0.3–2.4)	0.74	1.0 (0.5–1.9)	0.89	2.2 (0.9–5.1)	0.07
8–11	5.4 (1.8–15.8)	0.002	0.6 (0.2–2.0)	0.43	0.8 (0.4–1.9)	0.69	1.6 (0.7–3.2)	0.24
0–7	5.5 (1.9–16.3)	0.002	0.5 (0.2–1.8)	0.32	1.0 (0.4–2.5)	0.97	0.7 (0.3–1.5)	0.35
**History of contact with confirmed cases**	4.2 (2.6–6.9)	<0.001	3.8 (2.2–6.6)	<0.001	2.5 (1.7–3.5)	<0.001	1.0 (0.7–1.6)	0.9
**clinical symptoms (comparator: Non)3.5 (2.2–5.5)**								
1–2	6.6 (4.1–10.6)	<0.001	3.5 (2.2–5.5)	<0.001	3.3 (2.4–4.6)	<0.001	2.0 (1.6–2.6)	<0.001
≥3	10.8(6.2–18.9)	<0.001	2.5 (1.3–4.7)	0.004	6.2 (4.4–8.9)	<0.001	2.1 (1.5–2.8)	<0.001
**Co morbidities (comparator: Non)**								
1–2	1.0 (0.6–1.7)	0.87	1.8 (1.1–3.0)	0.01	2.3 (1.7–3.1)	<0.001	1.0 (0.8–1.3)	0.81
≥3	1.7 (0.7–4.1)	0.27	1 (0.2–4.2)	0.98	1.5 (0.7–3.1)	0.31	0.9 (0.5–1.7)	0.85

*88 cases in Group IV were detected positive using rapid antigen test.

## Discussion

During the early stages of the pandemic [from March to August 2020], schools were closed, and all instruction was switched to online delivery, with teachers only working from home. This was done because the higher authorities in Qatar were most concerned with containing and preventing the spread of the SARS-CoV-2 infection in the school environment. At the start of the subsequent academic year in 2020–2021, the requirement to reopen schools then materialized. Families are under a lot of financial, emotional, and physical strain, and Qatar values human interaction, therefore, they permitted openings with full in-person attendance at certain times [1–7]. We also noticed in our study that only 3.49% of the schools’ staff tested positive before opening the schools, and most of them were men and of older age, which was in accordance with the distribution of COVID-19 in the community. This was due to logical instruction pre-enrollment PCR testing for all staff, strict use of masks, physical distancing, and contact surveillance for any detected cases that helped reduce infection during the early stages of the pandemic [17,18]. We and others saw that the prevalence of new infections was low once the school opened, and there were no significant outbreaks during this time [6,14]. Before the school opened, the existence of symptoms, male gender, low level of education, and a history of contact with an infected case were all substantially linked to infection. These factors may account for the reduced prevalence of SARS-CoV-2 infection among instructors, especially given that the majority of them were female, lived in homes with families, and were being sampled five months after Qatari schools had closed. These numbers support our initial report on school personnel [14]. Before the B.1.1.7, UK variant emerged in Qatar after the school opened, the rate of infection did not rise, and only 3.39% of staff members, many of whom were young, contracted the illness at that time. Following the start of the school year, infection was substantially correlated with a history of contact with a proven infected case, the presence of symptoms, and the presence of comorbidities. The information is consistent with past research that did not identify a high infection rate among school personnel [12–14,19]. We tracked this population during the B.1.1.7 UK variant infection for additional investigation of the dynamics of viral transmission, and we discovered that the prevalence of infection rises to 7.34% but does not surpass that among the Qatari community [16,18] and we identified one reinfection. Young age and infant age following the start of the school, B.1.1.7 UK variant infections were substantially correlated with a history of contact with a confirmed infected case, shared housing, the existence of symptoms, and the presence of comorbidities. We estimated the Omicron infection that manifested later and discovered that the infection climbed to the maximum and reached 12.65% after this mass immunization program targeting school workers in Qatar began. Omicron -variant infection was substantially related to male gender, employment in the service sector, and the occurrence of symptoms. Our findings are consistent with earlier national reports that vaccination can lessen the severity of infection but cannot completely prevent Omicron infection, particularly since most cases were vaccinated at the time and 25 (0.77%) cases experienced reinfection. Most cases, apart from 6.1%, received vaccination, and breakthrough infection after vaccination occurred in 14.7% of cases [20–23].

### Conclusion

As a result, our analysis offers important new information about the spread of SARSCoV-2 among Qatari school employees from the start of the pandemic until the commencement of the Omicron wave in February 2022. The low rate of RT-PCR positives indicates that school screening and immunization programs are quite successful. The most crucial strategies for future pandemic control will continue to be the use of strict masks, social segregation policies, hygiene recommendations, and initiatives to find and isolate new cases of infection and their contacts. This will allow students, teachers, and their families to attend school in a safe environment without the need for additional strict lockdowns.

### Study limitations

Our study’s use of PCR testing alone may not be reflective of the prevalence overall, particularly in the early stages of the pandemic, when most cases are asymptomatic. But consistent monitoring of the same cohort both before and after the school’s opening offers a precise and unmistakable picture of infection dynamics. We did not evaluate the student infection rates, which is another drawback. According to national surveillance data, there was no increase in infection rates among kids who were old enough to attend school during that time. In addition, previous reports have indicated low incidence of infection among children [13,19]. As a result, definitive conclusions cannot be made because this population is not routinely screened.

## Supporting information

S1 File (XLS)
