# Correction: SARS-CoV-2 infection in 3,241 School working staffs: Impact of SARS CoV-2 variants of concern [Wild, B.1.1.7 and Omicron]

**DOI:** 10.1371/journal.pone.0326891

**Published:** 2025-06-20

**Authors:** Moza Alishaq, Jameela Ali Al Ajmi, Mohammed Shaheen, Mohamed Elgendy, Suni Vinoy, Anil George Thomas, Sam Joseph, Tintu Elizabeth Mathew, Renjith Joseph, Christymol Thomas, Anju K. Alex, Bincy Thomas, Asmaa Nafady, Hamed Elgendy, Hanaa Nafady-Hego

An additional affiliation is missing for the 14^th^ author. Hamed Elgendy is also affiliated with the Faculty of Medicine, Anesthesia Department, Assiut University, Assiut, Egypt.
